# Autonomic nervous system modulation during self-induced non-ordinary states of consciousness

**DOI:** 10.1038/s41598-023-42393-7

**Published:** 2023-09-22

**Authors:** Victor Oswald, Audrey Vanhaudenhuyse, Jitka Annen, Charlotte Martial, Aminata Bicego, Floriane Rousseaux, Corine Sombrun, Yann Harel, Marie-Elisabeth Faymonville, Steven Laureys, Karim Jerbi, Olivia Gosseries

**Affiliations:** 1 Sensation and Perception Research Group, GIGA Consciousness, University of Liège, Avenue de l’Hôpital 1, B34, 4000 Liège, Belgium; 2 Cognitive and Computational Neuroscience Lab, Psychology Département, University of Montréal, Montreal, Canada; 3 Interdisciplinary Algology Center, CHU, University Hospital of Liège, B35, Liège, Belgium; 4 Coma Science Group, GIGA Consciousness, University of Liège, Liège, Belgium; 5 TranseScience Research Institute, Paris, France; 6 Arsène Burny Center, Hospital of Liège University, Liège, Belgium; 7 Centre du Cerveau, Hospital of Liège University, Avenue de l’Hôpital 1, B34, 4000 Liège, Belgium; 8 CERVO Research Institute, Laval University, Quebec, Canada

**Keywords:** Neuroscience, Consciousness

## Abstract

Self-induced cognitive trance (SICT) is a voluntary non-ordinary state of consciousness characterized by a lucid yet narrowed awareness of the external surroundings. It involves a hyper-focused immersive experience of flow, expanded inner imagery, modified somatosensory processing, and an altered perception of self and time. SICT is gaining attention due to its potential clinical applications. Similar states of non-ordinary state of consciousness, such as meditation, hypnosis, and psychedelic experiences, have been reported to induce changes in the autonomic nervous system. However, the functioning of the autonomic nervous system during SICT remains poorly understood. In this study, we aimed to investigate the impact of SICT on the cardiac and respiratory signals of 25 participants proficient in SICT. To accomplish this, we measured various metrics of heart rate variability (HRV) and respiration rate variability (RRV) in three conditions: resting state, SICT, and a mental imagery task. Subsequently, we employed a machine learning framework utilizing a linear discriminant analysis classifier and a cross-validation scheme to identify the features that exhibited the best discrimination between these three conditions. The results revealed that during SICT, participants experienced an increased heart rate and a decreased level of high-frequency (HF) HRV compared to the control conditions. Additionally, specific increases in respiratory amplitude, phase ratio, and RRV were observed during SICT in comparison to the other conditions. These findings suggest that SICT is associated with a reduction in parasympathetic activity, indicative of a hyperarousal state of the autonomic nervous system during SICT.

## Introduction

Non-ordinary states of consciousness, such as hypnosis and meditation, have become the subject of increasing scientific study. While its definition can sometimes be elusive due to the variety of practices and states encompassed, certain psychological, phenomenological, and physiological features have been reported in the literature, gradually providing a framework for assessing key properties of non-ordinary state of consciousness^1–3^. Non-ordinary state of consciousness is distinguished from ordinary states of consciousness by its high level of absorption^4–6^, dissociation^2,7–9^, dereification, phenomenological reduction^10,11^, decentering^2,12,13^, cognitive diffusion^14^ or mindful attention^12^. Additionally, non-ordinary state of consciousness is known to modulate physiological systems such as the autonomic nervous system.

The autonomic nervous system is a division of the peripheral nervous system that controls involuntary bodily functions, helping to maintain homeostasis and respond to changes in the environment. It consists of the sympathetic and parasympathetic branches, which have opposing effects on various physiological processes such as heart rate, digestion, respiration, and pupil dilation^15^. The brain has evolved to regulate the internal environment of the organism by monitoring and anticipating needs^16^, integrating exteroceptive and interoceptive multimodal signals through an internal model of the world. This model constantly builds simulations and predictions based on prior experience and available energy resources^17,18^. During non-ordinary state of consciousness, similar mechanisms come into play, with interactions between ascending interoceptive signals and downstream responses through the autonomic nervous system being observed.

Heart rate variability (HRV) is monitored by descending autonomic nervous system control and reflects a self-regulatory mechanism through vagal modulation, as described by cardiac vagal control theory (CVC)^19,20^. Previous studies have shown modulation of HRV during non-ordinary state of consciousness^21,22^. Breathing, which can be modulated through voluntary control, also plays a role in the modulation of the autonomic nervous system^23,24^. Normal breathing exhibits a relatively constant rate and tidal volume, together constituting the normal respiratory rhythm. Variations within this rhythm are quantified and labeled as respiratory rate variability (RRV). Breathing can modulate HRV by increasing or decreasing heart rate, as well as through vagal mediation^25–27^.

Different non-ordinary state of consciousness practices utilize voluntary control of breathing to induce specific increases or decreases in HRV by manipulating breathing rate and variability. For example, practices like Samatha and Vipassana meditation or slow breathing techniques aim to increase HRV, while practices like holotropic breathwork aim to decrease it^28–32^.

However, not all non-ordinary state of consciousness practices involve the voluntary control of breathing. In some forms of meditation and hypnosis, increased vagal control has been observed without specific control over breathing^22,33,34^. Nevertheless, the literature shows some divergent effects depending on the specific technique used. For instance, certain types of meditation induce parasympathetic withdrawal (i.e., decreased HRV), while others produce the opposite effect (i.e., increased HRV)^35^. Previous studies have also shown that hypnosis induces a systematic increase in parasympathetic control^22,36,37^. These different physiological findings related to autonomic nervous system modulation in the context of non-ordinary state of consciousness may suggest relevant clinical opportunities.

In particular, drug-free self-induced and volitional non-ordinary states of consciousness are being increasingly tested and integrated into various clinical settings. These practices are employed in the treatment of mood disorders, pain reduction, and lead to improvement of quality of life^38,39^. Among these non-ordinary states of consciousness practices, self-induced trance states, which have historically received less attention, are believed to possess significant clinical potential. Trance states are often characterized along two dimensions: psychophysiological and cultural^40^. Various types of trance, such as hypnotic, shamanic, and possession trance, have been documented, and while they exhibit differences, they also share common phenomenological and psychological features^41,42^. Self-induced cognitive trance (SICT) has recently been introduced as a volitional non-ordinary state of consciousness. It is adapted from traditional Mongolian shamanic trance, abstracted from any ritual or spiritual expression^1,42^. SICT and shamanic trance are characterized by a lucid but narrowed awareness of external surroundings, a hyper-focused immersive experience of flow, expanded inner imagery, modified somatosensory processing, altered sense of self, subjective changes in time and space perception, modified body awareness, and cognitive and emotional states, along with disengagement from the sensory environment^1,43–47^. Unlike shamanic trance, SICT does not require the presence of a shaman to access the trance state. It is learned through a standardized training, initially using specific sound loops and later individualized movements or vocalizations that allow for deliberate and controlled induction of non-ordinary state of consciousness^41,42^. However, the neurophysiological and autonomic underpinnings of SICT remains unknown.

Autonomic regulation, along with several other physiological processes, plays a significant role in changes of consciousness states. Therefore, we hypothesized that SICT is associated with modulations of autonomic regulation. To test this hypothesis, we monitored HRV and breathing during SICT. The aim of this study is to investigate cardio-respiratory changes during SICT, with a specific focus on identifying the features among cardio-respiratory metrics that characterize the SICT state in comparison to a baseline resting state and an imagination control task condition.

## Methods

### Participants and training

We recruited a total of 27 adult participants who were native French speakers and had expertise in SICT. The participants had an average practice time of 32.6 months (± 50; min = 9; max = 216). It was a requirement for participants to be capable of remaining motionless during their trance. Data analysis was conducted on 26 subjects (with 23 females, mean age of 45 yrs, ± 13.33; min = 24; max = 72). One subject was excluded from the analysis due to excessive artifacts in the recorded data, such as movements and poor recording quality.

All participants received standardized training in SICT, which involved a system of techniques based on a sound loop developed by Corine Sombrun, an ethno-musician, and the TranceScience Research Institute (https://trancescience.org). This training program, inherited from shamanic Mongolian traditional practice, utilized the sound loop as a means for participants to enter into SICT. The objective of the training was to enable participants to voluntarily induce trance without the continuous use of sound. Following a training period consisting of two weekends of practice with this standardized program, most participants were generally able to induce trance voluntarily. Participants were then given the opportunity to continue practicing autonomously at home.

Prior to their participation in the study, participants were fully informed about the study's objectives and provided written consent. No incentives were offered and informed consent was obtained from all participants. The study received approval from the Ethics Committee of the Faculty of Medicine at the University of Liege and all experiments were performed in accordance with relevant guidelines and regulations.

### Procedure

Prior to the experiment, demographic data including age and gender were collected from each participant. The experimental session consisted of five conditions: ordinary conscious resting state ('Rest'), ordinary conscious state with auditory stimulations ('Auditory'), imagination task of a previous intense trance without entering a trance state ('Imag'), SICT, and SICT with the same auditory stimulations as the ordinary conscious state auditory condition ('Auditory-SICT'). The first three conditions were counterbalanced among participants, while the last two conditions were always conducted after the first three to avoid potential after-effects of SICT. The order of the last two conditions was consistent for all participants, starting with SICT without external stimulation, as it is commonly practiced in their daily life.

During each condition, participants were instructed to keep their eyes closed. In the Rest and Auditory conditions, they were asked to let their thoughts freely come and go. In the Imag condition, participants were instructed to imagine a previous intense SICT experience without actually entering a trance state. In the SICT and Auditory-SICT conditions, participants were instructed to induce and maintain SICT. The specific techniques used to induce SICT varied based on the participants' preferences and habits, involving body movements and/or vocalizations and lasting between 2 and 10 min. Once participants reached the trance state, they were instructed to remain motionless until the end of the recording. However, if the trance started fading away, participants were allowed to reinduce the trance and the recording was extended accordingly. Each condition had a duration of approximately 12 min.

Following the experimental procedure, participants completed a self-report to indicate whether they reached a trance state and rate the intensity of their experience on a Likert scale ranging from 0 (no trance) to 10 (the most intense trance ever experienced). It is important to note that this work excluded the two 'Auditory' conditions, as they are the focus of another related project. Therefore, the present study focused on three conditions: baseline resting state (Rest), imagination (Imag), and SICT.

### Electrophysiological recordings

During the experiment, we collected data on various body parameters, including electrocardiogram (ECG) and respiration using the EGI polygraph input box. The ECG data was obtained by placing two electrodes above and below the heart. For respiration monitoring, two belts were used, one placed around the chest and another around the belly. These measurements allowed us to capture physiological changes during the different experimental conditions and gain insights into the participants' physiological responses during the study.

### Data preprocessing and feature extraction

#### Cardiac analysis

For the cardiac analysis, the ECG time series data for each condition (Rest, Imag, and SICT) underwent preprocessing steps. The raw recordings were first cleaned by applying a high-pass Butterworth filter with a cut-off frequency of 0.5 Hz (order = 5). Powerline filtering was then performed to remove any interference from the electrical power source.

After the cleaning and preprocessing steps, 11 min of data (the longest duration available across all participants after removing the induction time) were selected for further analysis. R-peak detection was performed to locate the R peaks in the ECG signal, and the RR intervals (the time intervals between successive R peaks) were computed. Manual inspection was carried out to identify and correct any abnormal detections, such as aberrant RR intervals.

HRV features were extracted using Neurokit2, a Python package that provides advanced biosignal processing capabilities^48^. The following time domain features of HRV were computed: heart rate, mean of RR intervals (RR mean), standard deviation of RR intervals (SDNN), root mean square of the standard deviation of RR intervals (RMSSD), and the probability of RR intervals greater than 50 ms (pNN50). Frequency domain analysis was performed by applying the Welch method to compute the power spectral density of the RR intervals. The low frequency band (LF, 0.04–0.15 Hz) and the high frequency band (HF, 0.15–0.4 Hz) were extracted. Normalized powers (nLF, nHF) were obtained by dividing the power in a given frequency band by the total power. The logarithmic transformation of HF (lnHF) was also computed, along with the LF/HF ratio. Nonlinear metrics of HRV were assessed using the approximate entropy measure (ApEn), which quantifies the complexity of the HRV time series.

These preprocessing steps and feature extraction procedures allowed for the characterization of various cardiac parameters and HRV indices during the different experimental conditions (Rest, Imag, and SICT). Table 1 provides additional details about the extracted features and their interpretation.

**Table 1:** Heart Rate Variability (HRV) time and frequency domain metric, definition, and physiological mechanisms (adapted from Berntson et al., 1997; Laborde et al., 2017; Malik, 1996; Shaffer & Ginsberg, 2017).

		Name		Definition		Physiological mechanism	
Time domain		*SDNN*		Standard deviation of all RR intervals		Cyclic components responsible for heart rate variability	
		*RMSSD*		Root mean square of successive differences		Cardiac vagal activity	
		*pNN50*		Percentage of successive normal sinus RR intervals more than 50 ms		Cardiac vagal activity	
		*ApEn*		Approximate Entropy of RR intervals			
Frequency domain		*LF*		Low frequencies (0.04–0.15 Hz)		Mix of cardiac sympathetic and vagal activity, as well as baroreflex activity	
		*HF*		High-frequencies (0.15–0.4 Hz)		Cardiac vagal activity for ms2 only	
		*LF/HF*		Low frequencies/high-frequencies ratio		Unclear, suggested mix of cardiac sympathetic and vagal activity	
		*LFn*		Low frequencies divided by the total power			
		*HFn*		High frequencies divided by the total power			
		*LnHF*		Logarithmic transformed HF		Cardiac vagal activity	

#### HRV correction

In the HRV literature, several authors have highlighted the influence of heart rate and respiration rate on HRV metrics^30,49–52^. To account for the correlation between heart rate and HRV, we performed a correction on the HRV frequency domain metrics. Specifically, we multiplied each frequency metric by the mean RR interval for all conditions, as described by Sacha (2014). Following this correction, no significant dependence (p > 0.05) between HRV and heart rate, as well as between HRV and respiration rate, was found using correlation Pearson test.

#### Phasic and tonic HRV

According to the vagal reservoir theory (Laborde et al., 2018b), HRV should be assessed not only at rest but also in response to a task or during a particular state (such as effort or stress). The ability to modulate HRV during a task compared to its resting state is referred to as phasic HRV. To compute phasic HRV, we subtracted the values of SICT minus Rest and Imag minus Rest conditions as follows:

$${\text{HRVphasic }}\left( {{\text{Imag}}} \right) \, = {\text{ HRV}}\left( {{\text{Imag}}} \right) \, - {\text{ HRV}}\left( {{\text{Rest}}} \right)$$

$${\text{HRVphasic }}\left( {{\text{SICT}}} \right) \, = {\text{ HRV}}\left( {{\text{SICT}}} \right) \, - {\text{ HRV}}\left( {{\text{Rest}}} \right)$$

Subsequently, Spearman correlations between tonic HRV (Rest condition) and phasic HRV across participants were computed specifically for the HF HRV metric.

#### Respiration analysis

Similar to the cardiac signal analysis, we applied a similar procedure to analyze the respiratory signal. Respiratory time series were extracted for 11 min for each condition and each participant. The respiratory signal underwent preprocessing, including artifact cleaning (linear detrending followed by a fifth-order 2 Hz low-pass IIR Butterworth filter), and preparation for breath peak detection.

Using Neurokit2^48^, we extracted features from the preprocessed respiratory signal. The mean respiratory rate and amplitude were computed, along with the inspiratory to expiratory time ratio or Phase Duration Ratio. To compute the BB interval (interval between breath peaks), we located the B peak corresponding to exhalation onsets. Similar to the RR interval for the cardiac signal, every BB time series was manually checked and corrected for abnormal detection, such as aberrant BB intervals.

For the RRV analysis, we computed time domain features including the standard deviation of BB interval (SDBB) and the root mean square of the standard deviation of BB interval (RMSSD). These metrics provide insights into the variability of breath-to-breath intervals and reflect the dynamic nature of the respiratory system.

### Machine learning

Cardiac and respiratory signal classifications were performed using a linear discriminant analysis model within a leave-one-subject-out cross-validation scheme. The goal was to assess the discriminative changes in HRV and RRV between the Rest, Imagination, and SICT conditions.

In this approach, the model was trained on n − 1 subjects (25 individuals) and evaluated on the remaining subject. This process was repeated until each subject had been left out, and performance metrics were averaged across these repetitions. Three distinct classifications were conducted: Rest vs. SICT, Rest vs. Imagination, and SICT vs. Imagination. The purpose of these classifications was to characterize the differences between each condition.

Each classification was performed using a single feature at a time, utilizing the 27 features (16 cardiac metrics and 11 respiratory metrics) extracted from the data. The performance of the classification was assessed based on the decoding accuracy (DA), which represents the percentage of correctly classified observations in the test set.

To determine the statistical significance of the obtained decoding accuracy, permutation tests were applied. This involved generating a null distribution of decoding accuracies by randomly shuffling class labels and running multiple instances of the classification (n = 1000). By comparing the observed decoding accuracy to the null distribution, a statistical threshold was derived to assess significance. Maximum statistics were applied to control for multiple comparisons across all the metrics, following the methods described by^53–55^.

## Results

### Self-induced cognitive trance behaviors

All participants reported being able to reach a trance state in the SICT condition, with a reported mean intensity of 6.69 (sd = 1.89; min = 3; max = 10). The mean time of induction was 3.03 min (min = 1; max = 5).

### Time and frequency domain of HRV

**Figure 1:**
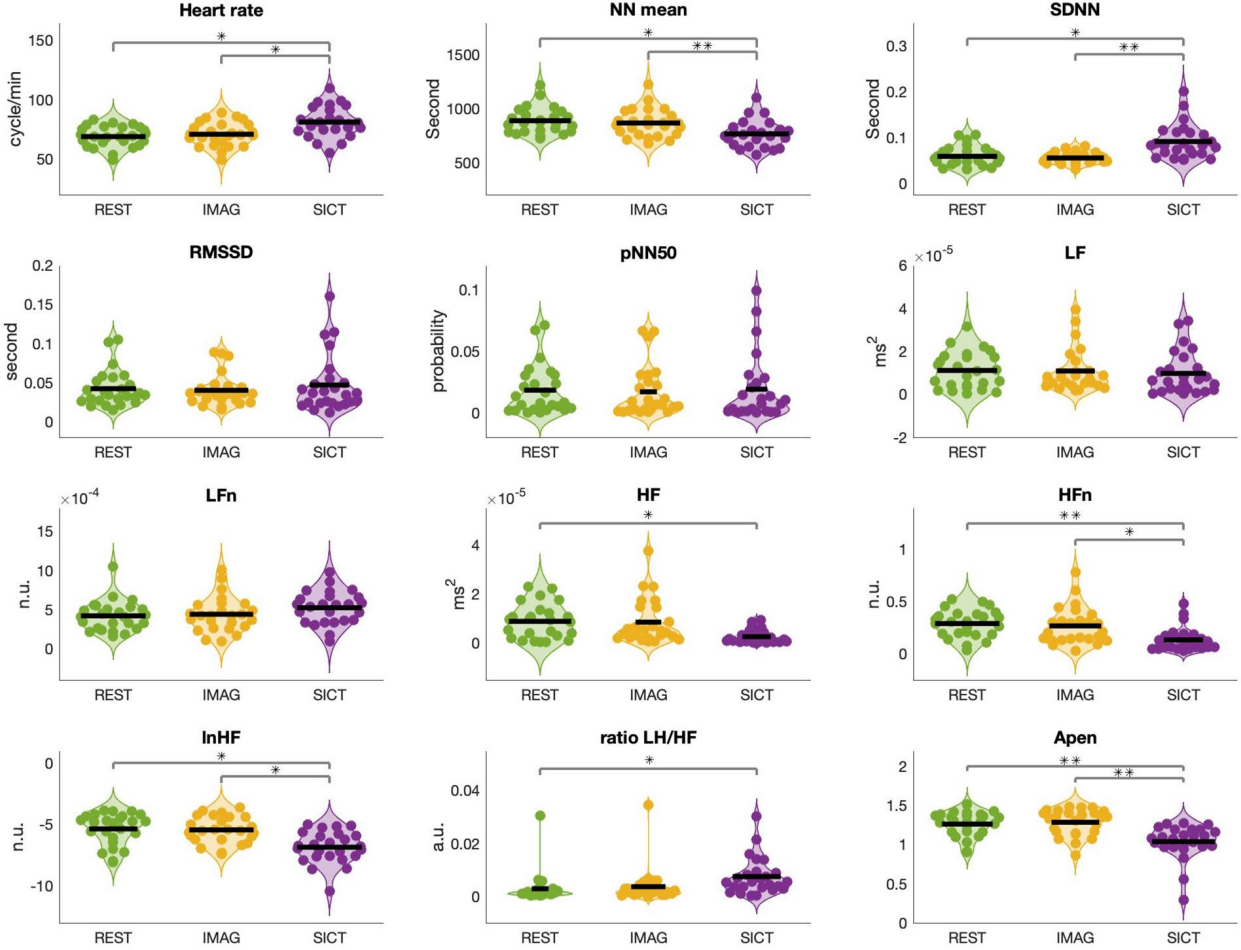
HRV time and frequency domain during Rest (green), Imagination (Imag, yellow) and self-induced cognitive trance (SICT, purple). *SDNN* standard deviation of RR interval, *RMSSD* the root mean square of standard deviation of RR interval, *pNN50* the probability of RR interval greater than 50 ms, *LF* low frequency, *HF* high frequency, *LFn* low Frequency normalized, *HFn* high Frequency normalized, *lnHF* logarithmic transformed HF, *ApEn* approximate entropy (corrected for multiple comparisons, *p = 0.05; **p = 0.01).

We observed an increase in heart rate during SICT (mean = 81.07 ± 12.53, standard deviation), compared to Rest (69.25 ± 8.67; DA = 71.9%) and between SICT and Imag (71.2 ± 9.88; DA = 68%) while no difference was found between Rest and Imag (DA = 56%). RR mean interval was lower in SICT (764.82 ± 121.8) compared to Rest (884.16 ± 120.64; DA = 69.9%) and also compared to Imag (862.27 ± 128.38; DA = 71.9%) while no significant difference was found between Rest and Imag (DA = 52%) (Fig. 1).

#### HRV time domain

SDNN was higher in SICT (67.45 ± 28.08) compared to Rest (51.57 ± 17.37; DA = 69.9%) and also compared to Imag (47.43 ± 12.75; DA = 76%) while no difference was found between Rest and Imag (DA = 50%). No difference was found for RMSSD between SICT (34.53 ± 25.30) and Rest (37.40 ± 19.57; DA = 50%) nor between SICT and Imag (34.63 ± 18.06) (DA = 52%), nor between Rest and Imag (DA = 52%). No difference was found for pNN50 between SICT (13.45 ± 17.68) and Rest (16.59 ± 17.06) (DA = 41.9%), nor between SICT and Imag (14.94 ± 18.23) (DA = 47.9%), nor between Rest and Imag (DA = 52%).

#### HRV frequency domain

**Figure 2:**
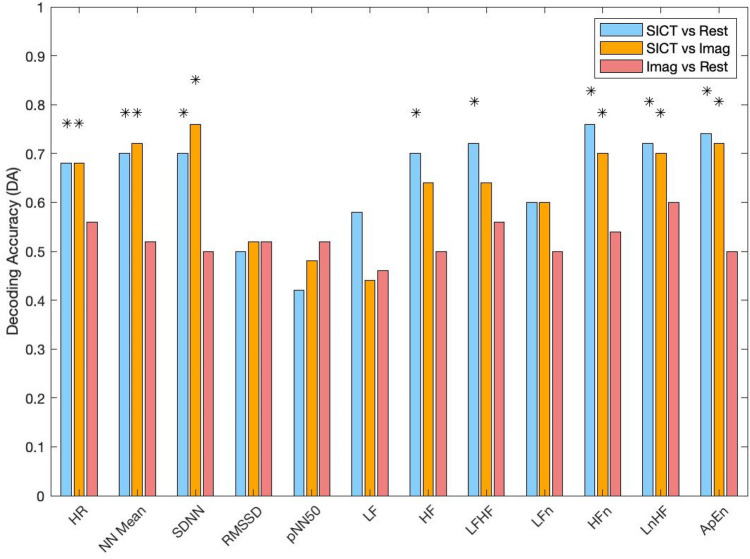
Decoding accuracy for HRV time and frequency domain during Rest, Imagination (Imag) and self-induced cognitive trance (SICT) between two class problem by linear discriminant (LDA) classifier; *: 5% across maximum statistic distribution.

We found a decrease for HF in absolute power (ms^2^) between SICT (0.0020 ± 0.0020) and Rest (0.0079 ± 0.0020; DA = 70%) but no statistical difference between SICT and Imag (0.0070 ± 0.0069; DA = 64%) nor between Rest and Imag (DA = 50%). We reported a decrease for HFn between SICT (0.13 ± 0.11) and Rest (0.29 ± 0.13; DA = 76%) and between SICT and Imag (0.26 ± 0.17; DA = 69%) while no difference was found between Rest and Imag (DA = 54%). A decrease for lnHF between SICT (−6.80 ± 1.28) and Rest (−5.28 ± 1.32; DA = 71.9%) and between SICT and Imag (−5.45 ± 1.05; DA = 69.9%) were found while no difference was found between between Rest and Imag (DA = 59%). LH/HF ratio was found to be increased during SICT (5.59 ± 5.18) compared to Rest (2.39 ± 4.68; DA = 71.9%) and compared to Imag (3.16; ± 5.65; DA = 64%), as well as an increase in Rest compared to Imag (DA = 56%). We found a drop in ApEn when comparing SICT (1.04 ± 0.21) to Rest (1.25 ± 0.15; DA = 73.9%) or to Imag (1.28 ± 0.16; DA = 68%) while no difference in ApEn was found between Rest and Imag (DA = 50%). We did not find statistical difference for LF and LFn across conditions (Fig. 2, Supplementary Information).

### Respiration rate, amplitude, phase and variability

**Figure 3:**
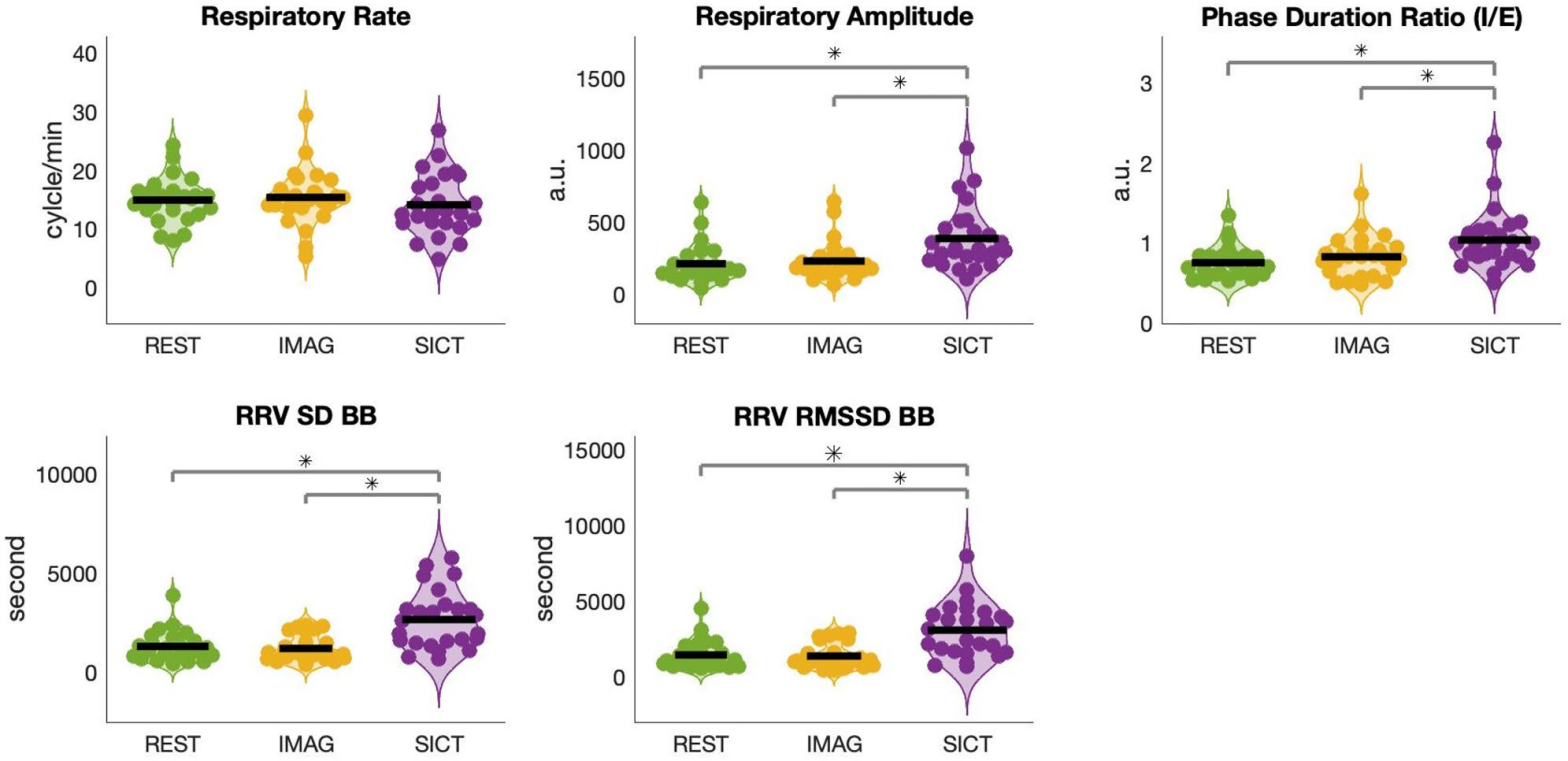
Comparison of thoracic respiratory rate, amplitude, phase and variability during Rest (green), Imagination (Imag, yellow) and Self-Induced Cognitive Trance (SICT, purple). *RRV* respiratory rate variability, *SD BB* standard deviation of BB (breath to breath) interval. *RMSSD* the root mean square of standard deviation of BB interval, *a.u* arbitrary unit, *I/E* inspiration/expiration (corrected for multiple comparisons, *p = 0.05; **p = 0.01).

**Figure 4:**
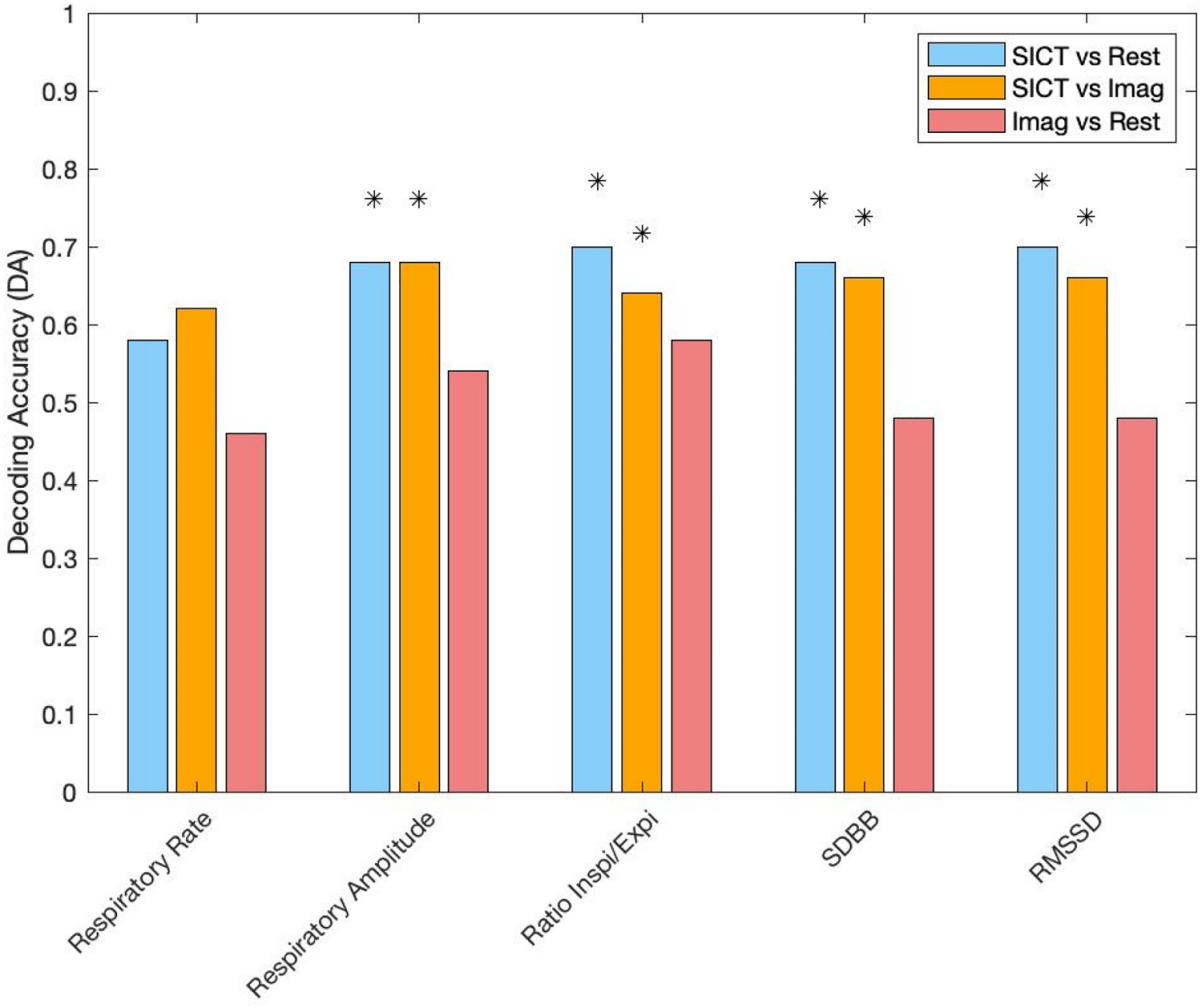
Decoding accuracy for respiratory features during Rest, Imagination (Imag) and self-induced cognitive trance (SICT) between two class problem by linear discriminant classifier; *: 5% across maximum statistic distribution.

Thoracic respiration rates showed a trend to decrease during SICT but no significant difference was found across all conditions, SICT (14.96 ± 6.54) compared to Rest (15.41 ± 4.38; DA = 58%), SICT compared to Imag (15.63 ± 4.80; DA = 62%) and Imag compared to Rest (DA = 46%) (Figs. 3 and 4). Respiratory amplitude increased during SICT (374.89 ± 225.53) compared to Rest (209.86 ± 134.01; DA = 68%) and between SICT compared to Imag (226.38 ± 136.33; DA = 68%) while no difference was found between Rest and Imag (DA = 54%). The phase ratio duration between inspiration and expiration was increased between SICT (1.04 ± 0.36) compared to Rest (0.74 ± 0.2; DA = 70%) and between SICT and Imag (0.83 ± 0.25; DA = 64%), while no difference was found between Rest and Imag (DA = 58%).

Respiratory rate variability changed across conditions. An increase in standard deviation (SD) was found between SICT (2.60 × 10^3^ ± 1.48 × 10^3^) compared to Rest (1.32 × 10^3^ ± 0.73 × 10^3^; DA = 68%) and between Imag (1.2 × 10^3^ ± 0.65 × 10^3^) and SICT (DA = 66%), while no difference was found between Rest and Imag (DA = 48%). The root mean square of standard deviation (RMSDD) was higher during SICT (3.01 × 10^3^ ± 1.77 × 10^3^) compared to Rest (1.44 × 10^3^ ± 0.92 × 10^3^; DA = 70%) but also compared to Imag (1.35 × 10^3^ ± 0.81 × 10^3^; DA = 66%), while no difference was found between Rest and Imag (DA = 48%).

### Phasic and tonic vagal cardiac control

**Figure 5:**
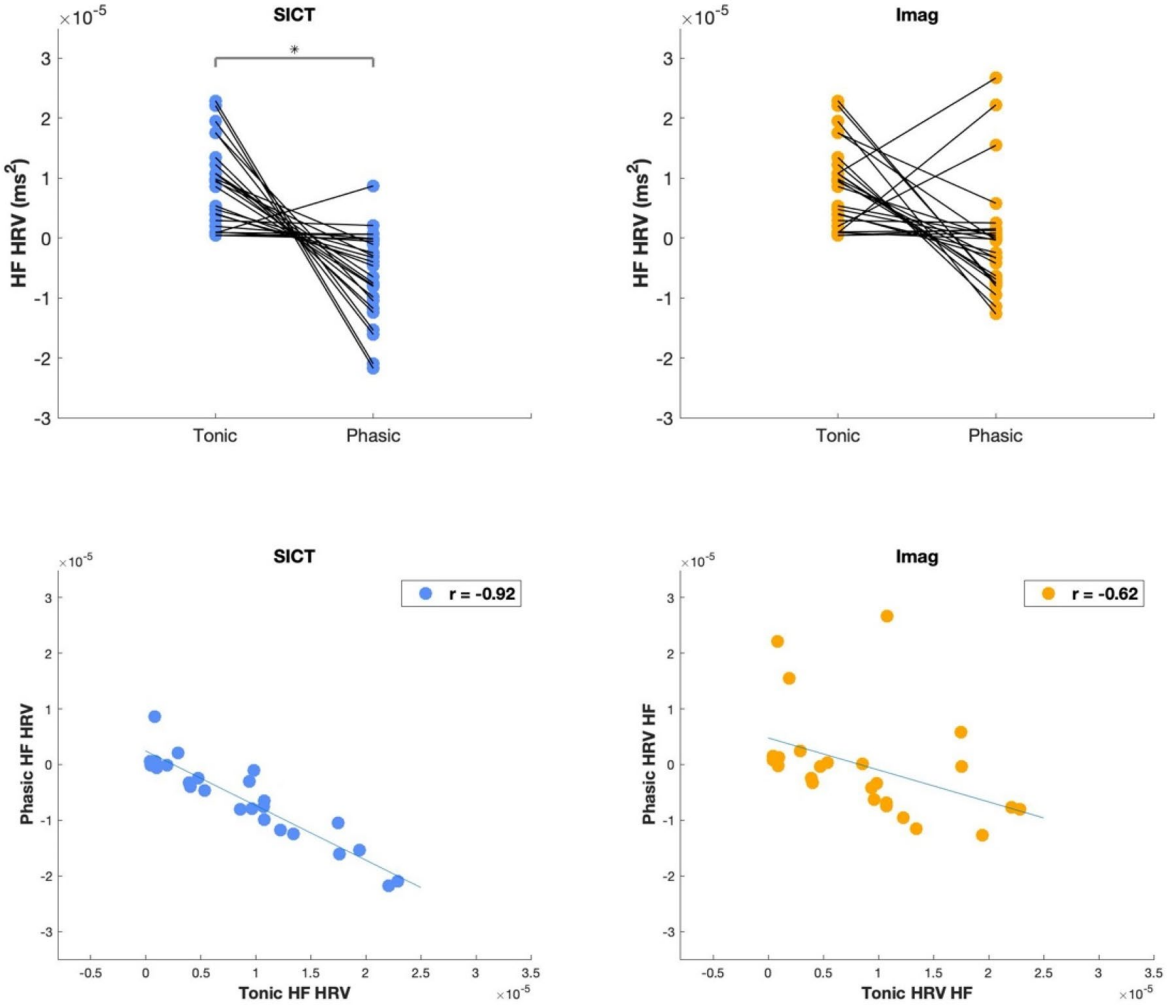
Individual difference and correlation between phasic and tonic HF during SICT and Imag conditions. Upper panel shows Tonic (i.e., Rest) and Phasic HF (i.e., HF-SICT minus HF-Rest or HF-Imag minus HF-Rest). Lower panel shows Spearman correlation between phasic and tonic HF for both conditions (SICT and Imag); *HF* high frequency, *p = 0.05.

We found that phasic HF during SICT decreased compared to tonic HF while in the Imag condition no difference were observed (Fig. 5). We also found significant spearman correlations across participants between phasic and tonic during SICT for HF (r = −0.92); while the same correlation between phasic and tonic HF during Imag was less strong (r = −0.62) (Fig. 5).

## Discussion

The objective of this study was to examine the physiological mechanisms of autonomic nervous system underlying SICT, focusing specifically on heart rate and breathing modulation during this non-ordinary state of consciousness. We compared the cardio-respiratory changes between ordinary consciousness states (resting state and imagination) and SICT. Our findings revealed significant differences in HRV and respiratory variability during SICT compared to the control conditions. Specifically, SICT was associated with an increase in heart rate, an increase in HRV time domain, a decrease in HF in the frequency domain of HRV, and a decrease in the entropy of the cardiac signal (ApEn). These changes were also characterized by a phasic decrease in HF-HRV. Additionally, respiratory changes indicated an increase in respiratory amplitude and variability. Importantly, no significant differences were observed between the imagination and resting state conditions.

These changes suggest a parasympathetic modulation of the autonomic nervous system, referred to as a vagolitic effect^27^, which implies an inhibition of the vagus nerve. The vagus nerve is the primary nerve of the parasympathetic nervous system, consisting of 80% afferent sensory fibers and 20% efferent motor fibers^27,56^. The efferent part of the vagus nerve that affects the heart is known as CVC and can be directly monitored through HF^57^. CVC is associated with a wide range of positive outcomes related to executive functions, emotion, and health, indicating overall better self-regulation of the organism^57,58^.

The observed withdrawal of HF during SICT is surprising, as it contrasts with a significant body of research demonstrating the detrimental effects of HF reduction. Decreased HF is considered predictive of poorer health outcomes, increased risk of cardiovascular disease and mortality^59^, chronic stress^60^, depression^61^, phobia^62^, schizophrenia and post traumatic syndrome disorders^63^. It has even been proposed that HF could serve as a psychopathology biomarker related to the autonomic nervous system^64^.

Our data support a withdrawal of HF during SICT, indicating a modulation of the nervous system associated with a decrease in parasympathetic activity. This modulation is observed from the resting state and continues during the task condition of SICT. These results can be interpreted in light of the *vagal tank theory*^20^, which suggests that self-regulation is based on vagal control at rest (also known as tonic CVC). This theory complements the *neurovisceral integration theory*^57^ by adding the mechanism of self-regulation, which involves how vagal control responds to specific events or tasks (reactivity, also called phasic CVC) and how the system returns to normal after the event (recovery). CVC withdrawal is associated with responding to environmental demands, including metabolically demanding states such as exercise, stress, attention, and information processing^27^.

Now, one might wonder why there is also a withdrawal of CVC observed during the imagination condition. According to the vagal tank theory^20^, one would have expected this effect to be specific to SICT since only this condition involves an environmental demand. These results could be attributed to the cognitive load required to induce imagination. This finding is consistent with the scientific literature conducted within the framework of the vagal tank theory, which suggests that self-regulation during tasks requires increased executive cognitive demands, resulting in a lesser decrease or even an increase in vagal control^19,20^. Furthermore, in our study, no significant difference was found between the imagination and resting state conditions. This result suggests that the recall of a memory does not recruit the same physiological mechanisms or intensity at the autonomic nervous system level compared to the experience of SICT itself.

During SICT, we also observed a consistent and larger decrease in phasic CVC compared to the other two control conditions. Previous studies have found a decrease in CVC during the fight-or-flight stress response, which led to near complete vagal withdrawal^27,65^. Therefore, a possible interpretation of our results is that this parasympathetic withdrawal is associated with an acute stress response. We also found an increase in amplitude and RRV during SICT, which could also be associated with an acute stress response. However, there is an inconsistency related to the trend of decreasing respiratory rate, as both an increased rate and tidal volume are markers of the acute respiratory response to stress^66^.

However, unlike a stress response, SICT is induced and can be stopped by will. Studies on inter-individual differences in physiological stress regulation have shown that a decrease in HRV is associated with an increase in stress-induced cortisol^67^. A plausible hypothesis is that regular practice of autonomic nervous system modulation (i.e., increase or decrease of CVC) could lead to improved autonomic regulation, as already demonstrated by regular physical activity (decrease of CVC) or regular mindfulness meditation (increase of CVC)^32,68^. Consequently, regular practice of SICT could enhance control over acute stress responses typically triggered by uncontrolled external stimuli (i.e., the detection and interpretation of danger). Developing the ability to manage and control these stress response mechanisms through willpower could be beneficial for various clinical populations affected by psychopathologies associated with dysregulation of the physiological stress system, such as anxiety and post-traumatic stress disorder.

The withdrawal of CVC could be a hyperactivation of the autonomic nervous system associated with a SICT-induced dissociation process, which is typical of other forms of non-ordinary states of consciousness^8,9,69^. Certain forms of intense stress have been associated with a dissociation process in humans dissociative experience that is physiologically linked to the autonomic nervous system through the limbic system, which, along with the frontal cortex, forms the neural basis of the depersonalization model^70–72^. In the literature, non-ordinary states of consciousness can be classified at the physiological level as either an increase in parasympathetic tone, corresponding to the relaxation model and also associated with hypnosis^73^ or certain types of meditation^31,74,75^ and ecstatic experience^40^, or a decrease in parasympathetic tone, as found in SICT and certain types of meditation^23,76^, and probably other types of trance^40^.

The effects of SICT on the autonomic nervous system found in this study are not consistent with the scientific literature on other forms of non-ordinary states of consciousness, such as meditation, which typically induces an increase in parasympathetic tone^36^. However, when comparing autonomic nervous system changes under SICT with other forms of non-ordinary states of consciousness, it is important to consider the techniques used, the type of induction employed, the associated psychological state, and the reported phenomenology under these states.

That said, some studies have shown varying effects of meditation on the autonomic nervous system^76^. For example, Amihai et al. (2014) compared two meditation styles (*Theravada* and *Vajrayana*) and found opposing results in terms of HF power Theravada meditation, which includes practices involving sitting, focusing on breath, and monitoring attention, showed an increase in parasympathetic tone associated with the intensity of the practice. In contrast, Vajrayana meditation, which includes practices of high absorption level, dance, and emotional experience, showed the opposite effect, i.e., a parasympathetic withdrawal, with a decrease in HF power, similar to what we observed in SICT in our results^76^. Many similarities can be drawn between Vajrayana and SICT states, not only at the physiological level but also at the phenomenological or behavioral level (body signs), including rich multimodal activity, sensory-motor patterns, feelings and emotions, as well as dereification, self-decentering, and an altered sense of self^76^. Integrative studies comparing the effects of various forms of non-ordinary consciousness on autonomic nervous system activity would, therefore, be necessary to confirm their differences and similarities from a neurobiological point of view.

### Limitations and future studies

Our study has several limitations that should be acknowledged. Firstly, the generalizability of our results is limited due to the small sample size and the imbalanced female-male ratio. Replication of our findings in a larger and more diverse population is necessary. Secondly, the naturalistic design of our study restricts the ability to make long-term predictions based on our findings. Future research should aim to investigate the long-term effects of SICT on autonomic nervous system activity in a more controlled setting. Thirdly, comparing long-term basal values with the phenomenological content and emotional content experienced during SICT would provide a more comprehensive understanding of the physiological impact of repetitive practices.

Furthermore, integrating interoceptive and brain data would be valuable in elucidating autonomic self-regulatory mechanisms. Future studies should explore the relationship between autonomic nervous system modulation and neuro-psychological interactions to better comprehend why a hyperarousal state of the autonomic nervous system can either contribute to or be indicative of psychological states such as dissociation or absorption. Assessing the activity of brain regions known to activate the physiological stress system, such as the amygdala, in association with SICT would be of interest. Additionally, evaluating the activity of the hypothalamic–pituitary–adrenal axis during SICT could further confirm the hypothesis of physiological stress system involvement. In summary, addressing these limitations and incorporating additional measures and analyses in future studies would enhance our understanding of the physiological and psychological aspects of SICT.

## Conclusion

This study is the first to provide insights into the physiological component of the autonomic nervous system during SICT. Our findings reveal that SICT is characterized by a decrease in cardiac vagal control, leading to a hyperarousal state of the autonomic nervous system. This physiological response bears resemblance to the stress response observed in other forms of non-ordinary states of consciousness, such as meditation, and may involve the autoregulatory systems of the cortex and limbic regions. The induction of SICT appears to be associated with alterations in the amplitude, phase duration, and variability of the respiratory rhythm. Overall, this research emphasizes the central role of the autonomic nervous system in SICT as an embodied experience. It invites further research investigations into the regular practice of SICT and its potential implications on the underlying physiological mechanisms of stress regulation.

### Supplementary Information

Supplementary Figure 1.

## Data Availability

The complete dataset is available upon reasonable request (avanhaudenhuyse@chuliege.be; ogosseries@uliege.be).

## Abbreviations

SICT: Self-induced cognitive trance
HRV: Heart rate variability
RRV: Respiration rate variability
CVC: Cardiac vagal control
DA: Decoding accuracy
