# Supplementary material for: Autonomic nervous system modulation during self-induced non-ordinary states of consciousness
Source: Sci Rep. 2023 Sep 22;13:15811. doi: 10.1038/s41598-023-42393-7 (PMC10516905; doi:10.1038/s41598-023-42393-7)
Supplement: Supplementary file 1 — Supplementary Figure 1. [file 41598_2023_42393_MOESM1_ESM.docx]

***Supplementary material***

Inter-Individual dynamic modulation of HRV during SICT

The successive mean of HFn value shows higher HFn higher during the Rest conditions and a decrease of HFn during induction, and during the whole SICT period. Median split based on Rest period shows that subjects who have higher HFn at rest shows greater decrease during SICT compared to subjects who have lower HFn at rest, and both median tend to converge to the mean during SICT.

*
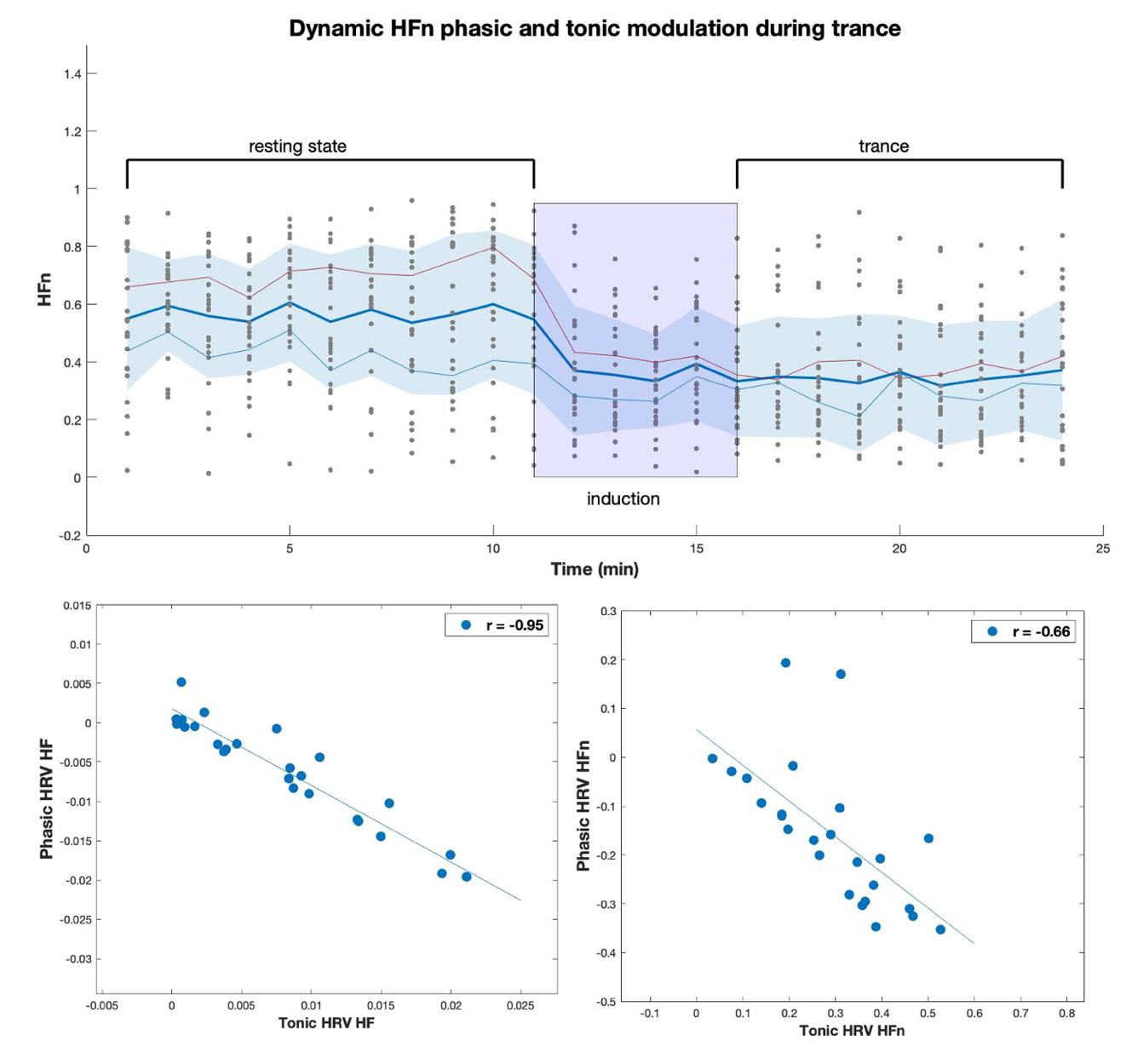
*

*Supplementary Figure 1 (SF-1): Inter-individual dynamic modulation between phasic and tonic cardiac vagal control during SICT.* *Upper panel shows dynamic variation in HFn mean for 11 min of resting state and 13 min of trance including induction (between 1 to 4 min depending on participants). Individual data and mean in bold blue line, red and blue line show mean in high (red) and low (blue) after a median split based on the resting period.*
